# An open ecosystem engagement strategy through the lens of global food safety

**DOI:** 10.12688/f1000research.6123.1

**Published:** 2015-05-27

**Authors:** Paul Stacey, Garin Fons, Theresa M Bernardo

**Affiliations:** 1 Creative Commons, Mountain View, CA, 94042, USA; 2 Center for Open Educational Resources and Language Learning (COERLL), University of Texas at Austin, Austin, TX, 78712, USA; 3 College of Veterinary Medicine, Michigan State University, East Lansing, MI, 48824, USA

**Keywords:** food safety, open educational resources, OER, open access, open data, open policy, Creative Commons, knowledge sharing

## Abstract

The Global Food Safety Partnership (GFSP) is a public/private partnership established through the World Bank to improve food safety systems through a globally coordinated and locally-driven approach. This concept paper aims to establish a framework to help GFSP fully leverage the potential of open models.

In preparing this paper the authors spoke to many different GFSP stakeholders who asked questions about open models such as:

- what is it?
- what’s in it for me?
- why use an open rather than a proprietary model?
- how will open models generate equivalent or greater sustainable revenue streams compared to the current “traditional” approaches?

This last question came up many times with assertions that traditional service providers need to see opportunity for equivalent or greater revenue dollars before they will buy-in. This paper identifies open value propositions for GFSP stakeholders and proposes a framework for creating and structuring that value.

Open Educational Resources (OER) were the primary open practice GFSP partners spoke to us about, as they provide a logical entry point for collaboration. Going forward, funders should consider requiring that educational resources and concomitant data resulting from their sponsorship should be open, as a public good. There are, however, many other forms of open practice that bring value to the GFSP. Nine different open strategies and tactics (Appendix A) are described, including: open content (including OER and open courseware), open data, open access (research), open government, open source software, open standards, open policy, open licensing and open hardware. It is recommended that all stakeholders proactively pursue "openness" as an operating principle.

This paper presents an overall GFSP Open Ecosystem Engagement Strategy within which specific local case examples can be situated. Two different case examples, China and Colombia, are presented to show both project-based and crowd-sourced, direct-to-public paths through this ecosystem.

## Introduction

The Global Food Safety Partnership (GFSP) is a public/private partnership established through the World Bank. Open models have the potential to significantly enhance the GFSP’s goal of improving food safety systems through a globally coordinated and locally-driven food safety approach. This open models concept paper aimed to establish a framework to help leverage that potential.

We explored a range of open models that could enhance the scalability and sustainability of food safety. Our primary goal was to show how open models could support GFSP’s efforts to help ensure safe food, increase food supply chain value, accelerate economic growth, alleviate rural poverty, and improve public health outcomes.

In developing open models the sub-working group considered the many stakeholders involved in global food safety including:

- governments
- regulatory agencies - public regulators, inspectors and managers
- private sector agri-food processors and manufacturers
- farmers and producers
- universities, service providers, trainers and certification bodies
- international organizations
- non-governmental organizations (NGOs)

Open models increase opportunities for access and participation so our models also identified new stakeholders including the general public.

We set out to define and design open models that generate impact and benefits from a multi-stakeholder perspective. Open models show how the many forms of openness, including such things as Open Educational Resources (OER), open access (OA), open data, and open policy can be adopted across all stakeholders and at different stages of knowledge production and dissemination. Open models provide a new paradigm for multi-stakeholder collaboration and capacity building.

Multi-stakeholder adoption of open models generates cumulative benefits for all stakeholders. The greater the number of stakeholders that use open models, the larger the impact. Going open also means that new stakeholders have the agency to get involved and participate. Open models in this paper show how both traditional and new stakeholders can collaborate in the use of open resources and practices to enhance global food safety.

New technologies offer opportunities for information sharing, public participation, and collaboration. A frequently cited benefit of openness is lower costs for funders and users ^i, ii^. Lower costs are amplified by using digital Information and Communication Technology (ICT) which, in combination with openness, creates opportunities for free, or very low cost, large scale access and participation. Open models harness these technologies to make more food safety information public and actively engage citizens in improving and disseminating food safety knowledge.

The primary goal of open models in this paper is to scale and disseminate food safety knowledge and practices to generate social and economic benefits. While open models can be adopted alongside status quo operations, preserving existing business models and traditional revenue streams were not the priority. Open models often disrupt traditional practices and business models. The open models outlined in this paper involve alternative business model approaches for all stakeholders.

In preparing this paper the open models sub-working group spoke to many different GFSP stakeholders. Underlying many of those conversations were questions about open models such as:

- what is it?
- what’s in it for me?
- why use an open model rather than a proprietary model?
- how will open models generate equivalent or greater sustainable revenue streams compared to the current “traditional” approaches?

This last question came up many times with assertions that traditional service providers need to see opportunity for equivalent or greater revenue dollars in real terms before they will buy-in. This paper identifies open value propositions for food safety stakeholders and proposes a framework for creating and structuring that value. However, we recommend the GFSP not attempt to use open models and at the same time try and preserve the traditional business models of all partners. Open models enable access, scale, massive adoption, lower costs, localization and social networks, but only if existing business models are set aside and new ones adopted. Traditional models are resistant to open innovation. As a result open models are often more aggressively and strategically pursued by providers who adopt new open business models and play by different rules. Open models are not “business as usual”. Open models cannot be adopted and driven by questions like “Who pays?” assuming the same players and beneficiaries as in the traditional model. This is not to say that open models ignore the financial underpinnings of global food safety and the need for stakeholders to pay bills and keep the lights on. That is understood, but the economics of open models are different and preserving traditional business models is secondary to achieving global food safety goals. Open models are better framed by questions like “How much of this can be free?” and “Where can I add value?”.

Balancing calls for “show me the money” were aspirations for open models to improve food safety scalability and sustainability. We heard loud and clear the need for a macro, generic open model that depicts food safety as an ecosystem at the global and local level. This open models concept paper presents an overall GFSP Open Ecosystem Engagement Strategy within which specific local case examples can be situated. Two different case examples, China and Colombia, are presented to show both project-based and crowd-sourced, direct-to-public paths through this ecosystem.

OERs were the primary open practice GFSP partners spoke to us about, as they provide a logical entry point for collaboration. Going forward, funders should consider requiring that educational resources and the concomitant data resulting from their sponsorship should be open, in the same manner that publicly funded research (and more recently data) is available as a public good. There are, however, many other forms of open practice that bring value to the GFSP. This paper names and describes nine different open practices ( Appendix A) stakeholders can use to generate food safety value including: open content (including OER and open courseware), open data, open access (research), open government, open source software, open standards, open policy, open licensing and open hardware. It is recommended that the GFSP adopt as many of these open practices as possible, not just OER. Parallel to Metcalfe’s law which states that the value of a network is proportional to the square of the number of users, open model value becomes reciprocal and is magnified when a wide range of open practices are adopted by a large number of stakeholders. Overlaid on both the China and Colombia case examples are suggestions for which open practices can be adopted by which stakeholders.

This paper presents an overarching framework intended to guide GFSP partners in their thinking and adoption of open models. It does not get down into the specifics of identifying open business models and approaches for each individual stakeholder. However, this is a logical next step and is recommended as a follow-on for both global and local GFSP initiatives. For those interested in understanding the economics of open business models a short list of recommended books and readings is provided in Appendix B.

## An open ecosystem engagement strategy

**Figure 1.**
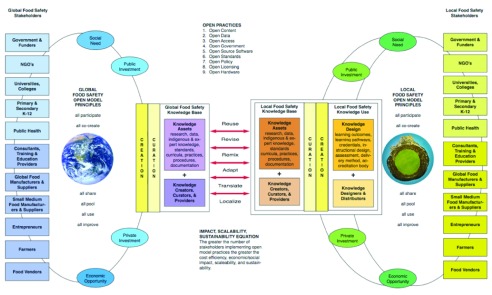
Open Ecosystem Engagement Strategy. On the left are global stakeholders who, driven by social needs and economic opportunities, are making public and private investments and creating a coordinated approach to improving food safety. To generate maximum value through open models all global stakeholders adopt open principles which have everyone participating, co-creating, sharing, pooling, using and improving. Nine different open practices can be adopted as a means of fulfilling those principles. Definitions for each of these nine open practices, examples of success, and sample stakeholder value propositions associated with them are in Appendix A at the end of this paper. Collectively this generates an open global food safety knowledge base made up of knowledge assets (e.g. text, graphics, sound, video, lists of experts and expert networks, etc.) and knowledge creators, curators and providers. Local food safety stakeholders are shown on the far right. While the categories are the same the actual organizations are different. To generate maximum value through open models all local stakeholders adopt open principles which have everyone participating, co-creating, sharing, pooling, using and improving. The same nine different open practices can be adopted as a means of fulfilling those principles. Collectively this generates an open local food safety knowledge base made up of knowledge assets and knowledge creators, curators and providers. Credit: The Blue Marble by NASA, Public Domain; Planeta Loarre by Juandc CC BY.

Figure 1 depicts food safety as an ecosystem at the global and local level and represents a macro overarching framework for open models. The open aspects of this work make it possible for GFSP to make use of both the global and local food safety knowledge bases. Global food safety knowledge can be reused, revised, remixed, adapted, translated, and localized for local food safety provision. And the reverse is also true, local food safety knowledge can be reused, revised, remixed, adapted, and translated into global resources.

Open model implementation of local food safety involves the formation of partnerships between global and local stakeholders who then design and distribute the knowledge in a way that meets local social and economic needs.

Impact, scalability, and sustainability can be thought of as an equation where maximum impact, scale, and sustainability are achieved by having the maximum number of stakeholders adopt the maximum number of open practices.

## Case Example 1: China

The global food system has changed dramatically as multinational supermarkets and their procurement channels have rapidly expanded into emerging markets. Consumers are demanding safe, high-quality food. In response, governments and industry are collaborating to assure quality and food safety consistently around the world. One area of focus has been the development of protocols and training for suppliers and people responsible for food safety compliance.

Starting in 2008, the Food Safety Knowledge Network (FSKN) a collaboration between Michigan State University (MSU), the Global Food Safety Initiative (GFSI) of the Consumer Goods Forum, and other food industry and public sector partners began strengthening the food industry’s response to the complex food safety knowledge and training challenges that affect emerging markets by providing free access to high-quality, standardized OERs ^iii^. These OERs, for basic and intermediate levels of food manufacturing, are based on competencies developed by the Consumer Goods Forum. Their Global Markets Working Group has defined company characteristics of suppliers which have been used by MSU to create OER and proprietary pre- and post-tests. These are also based on the global and country-specific standards.

GFSP's 5-year work program of demand-driven food safety capacity building and advisory services for low and middle income countries was preceded by an initial programming and preparatory year (2012) that included implementation of a training program developed in partnership with the Asia Pacific Economic Cooperation (APEC) and other partners, on food safety prerequisites and Hazard Analysis & Critical Control Points (HACCP) delivered in Beijing in June, 2012. This program was comprised of 3–4 weeks of online learning followed by a 6 day intensive face-to-face session (with real-time live translation) focused on skills development. More recently in the summer of 2013 a similar program was conducted in Shanghai.

These programs are making use of existing OERs, building on those developed by FSKN/MSU, in a range of formats from PowerPoint presentations to more narrative and full curricula developed in partnership with APEC and the World Bank. Content for the Basic Global Markets Training Program (Archived at http://www.webcitation.org/6Y28HOsqN) is now up to version 2 and version 3 China specific translations.

**Figure 2.**
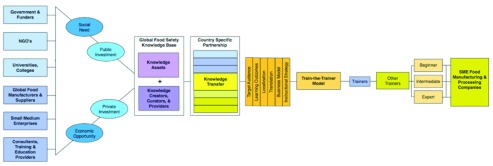
Case example of China. This figure provides an overview of collaborators, resources and approaches being implemented in China.

The cumulative build-out of this work has established a global food safety knowledge base made up of knowledge assets and knowledge experts as depicted in Figure 2.

GFSP’s current focus on China as a priority is building on this predecessor work. The main effort will be focused on generating economic growth by building out food safety knowledge and competencies of the estimated four hundred thousand food manufacturers and suppliers in China. The plan is to scale up use of existing open resources and roll out a program using a train-the-trainer approach in the fall of 2014.

The China work involves the formation of partnerships including:

- funders - such as United Nations Industrial Development Organization (UNIDO), Global Food Safety Partnership (GFSP), World Bank, International Finance Corporation and others
- nonprofits - U.S. Pharmacopeial Convention, Grocery Manufacturers Association Foundation
- universities - Shanghai Jiao Tong University

Additional China-based partners are still being established.

The business model for the China train-the-trainer program is to reuse existing open educational resources and make little to no upfront investment in training materials. Public investment is being sought to support the initial train-the-trainer delivery. Downstream delivery to food manufacturers and suppliers would entail participants paying a fee.

Scalability & sustainability challenges include:

- finding someone entrepreneurial to run this as a business
- establishing facilities and resources for needs assessment, logistics, registration, Learning Management Systems (LMS), etc.
- ensuring quality of trainers
- keeping the content up to date
- base OERs are country agnostic so need adaptation to fit country and sector needs including preventive control information and country specific requirements to meet local regulations.
- some partners want to do training using their own proprietary content
- assessment components of training are proprietary not OER
- downstream, the suppliers pay a fee for training to cover costs and improve materials, but must be affordable to suppliers
- after initial train-the-trainer in-country, partner must be responsible for continuous roll out and scaling up. Earlier initiatives have not scaled up as expected.
- need to move beyond the training – the model needs to incorporate mentoring and skills development and application beyond the initial training
- some partners need to make revenue from service provision
- need an open platform to coordinate the partnership, organize implementation, and reduce duplication of effort

Table 1 indicates the potential starting points for various stakeholder groups in China in using the nine different types of open practices. For example, government and funders are poised to make use of eight of the nine open practices (open content, open data, open access, open government, open sources software, open policy and open licensing), whereas the use of open hardware, which is a relatively new form of open practice, is most likely to be initiated by universities and colleges.

**Table 1.** Potential starting points for stakeholder use of open practices: China.

CHINA	1 Open Content	2 Open Data	3 Open Access	4 Open Government	5 Open Source Software	6 Open Standards	7 Open Policy	8 Open Licensing	9 Open Hardware
**Government/Funders**	x	x	x	x	x	x	x	x	
**Non-Governmental** **Organizations**	x		x				x	x	
**Universities/Colleges**	x	x	x		x	x	x	x	x
**Global Food** **Manufacturers &** **Suppliers / Small &** **Medium Enterprises**	x				x	x	x	x	
**Consultants, Training** **Providers, Educators**	x				x			x	

## Case example 2: Colombia

In Colombia the provincial government of Cundinamarca in partnership with Convenio Andres Bello and education partners are interested in open and distance education as a means of rural social development including food safety.

Convenio Andres Bello is an international intergovernmental organization including Bolivia, Chile, Colombia, Cuba, Ecuador, Spain, Mexico, Panama, Paraguay, Peru, Dominican Republic and Venezuela. It is led by the Ministers of Education of the member countries. Convenio Andres Bello promotes consensus building among members and joint action plans for culture, education, science and technology. Convenio Andres Bella's strategic plan focuses implementation of an ordered set of initiatives under four program areas:

1. The educational sector and the construction of citizenship
2. Social appropriation of knowledge and learning and citizenship
3. Sustainable development, climate change and citizenship
4. Policies: educational, cultural, scientific, technological and citizenship

Cundinamarca and Convenio Andres Bello have asked the OpenCourseWare Consortium (OCWC) for help in designing a digital learning initiative with a goal of adopting open online education in support of these aims. The OCWC is a worldwide community of hundreds of higher education institutions and associated organizations committed to advancing open education and its impact on global education. The consortium seeks to engender a culture of openness in education to allow everyone, everywhere to access the education they desire, while providing a shared body of knowledge and best practices that can be drawn upon for innovative and effective approaches. In addition the OCWC helps to solve social problems through expansion of access to education.

**Figure 3.**
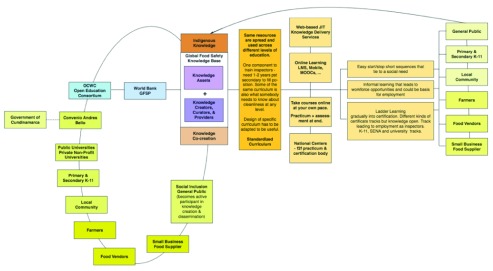
Case example of Colombia. This figure provides an overview of collaborators, resources and approaches being implemented in Colombia.

The OCWC is responding to this request by assembling a group of experts in open and online education to support a redesign project, and proposes to collaborate with the GFSP’s Learning and Knowledge Working Group to offer a curriculum, on a national scale in Colombia, in food safety leading to certification ( Figure 3).

The GFSP may benefit through the development of standardized curricula and courses that are aimed at different target groups including:

1. residents of impacted communities
2. technical institutes that may want to include curriculum in food safety aiming at employment as inspectors and other skilled professions
3. K-11 teachers for inclusion in primary and secondary health curricula, and
4. university departments for inclusion in undergraduate and graduate degree programs

The aim is to start with a substantial body of food safety knowledge already in OER form available through GFSP and other stakeholders. Combined with the existing local curriculum, the intent is to adapt these core resources to address different levels of education. A curriculum component that provides education on what someone needs to know about cleanliness at any level might also be a curriculum component for someone enrolled in a two year program to become a certified food inspector. OER will be adapted for the Colombian context and used to create:

- easy start/stop short sequences that tie to a social need
- informal learning that leads to workforce opportunities and could be basis for employment
- ladder learning that progressively builds gradually into certification.
- different kinds of certificate tracks including employment as inspectors as well as K-11, college, and university tracks

In addition to food safety pertaining to food manufacturing and supply, the Colombia program seeks to:

- situate food safety deep down in society - on the farm, in the home, in the local community, and use open models to make knowledge community based impacting people on the ground
- improve nutrition, reduce sickness
- achieve better learning through improved food nutrition
- have the learning lead to employment opportunities, and so reduce poverty
- provide food safety and security related to growing, harvesting, storing, and shipping food, as well as food safety pertaining to food preparation and cleanup

Curricula will be made open to all, enabling local communities, farmers, food vendors, small business food suppliers and even the general public to become active participants in knowledge creation and dissemination. This open model ( Figure 3) blends expert and indigenous knowledge into a knowledge co-creation open model. One ambitious concept is supporting a free path to certification – and employment – for members of communities most affected by food security and problems with food safety.

As part of its redesign for digital learning a full range of contemporary options are being considered including:

- online learning via LMSs
- Massive Open Online Courses (MOOCs)
- mobile technology

There also are opportunities for entrepreneurs to play a role in providing web-based, just-in-time knowledge delivery services (like iCow which provides timely information to small-holder cattle farmers).

Certification tracks will be designed in such a way that participants can take courses online at their own pace with a practicum and assessment at the end. National centers will be used for the face-to-face practicum and assessment with colleges and universities being the certification entities.

The Colombia case example has a unique business model. The concept is that the avoidance of public health problems that are caused by unsafe foods can more than pay for the costs of employment in the area of training, monitoring and reporting on food safety in poor areas, both rural and urban. In raising the profile of food safety in these communities, related issues of nutrition and food security can be included to support even better social results, including lowered health costs and improved educational results. You can save more money on not providing emergency services than what it would cost you to provide education through this open model. Savings generated through improved nutrition, public health, and reduced days lost to illness, clinic visits and use of medical facilities pay for the food safety education. Using an open model could make the cost of the education very low. Revenue will be generated from those seeking formal certification. However, programs targeted at displaced, impacted communities will be government funded or have a publicly subsidized lower fee.

A matrix of stakeholder groups in Colombia and their potential starting points in using the nine different types of open practices is presented in Table 2. For example, the OCWC makes use of open content, open access, open standards, open policy and open licensing. The community, farmers, entrepreneurs, food vendors and suppliers have the potential to make use of open content, open access, open source software, open standards and open hardware.

**Table 2.** Potential starting points for stakeholder use of open practices: Colombia.

COLOMBIA	1 Open Content	2 Open Data	3 Open Access	4 Open Government	5 Open Source Software	6 Open Standards	7 Open Policy	8 Open Licensing	9 Open Hardware
**OpenCourseWare** **Consortium**	x		x			x	x	x	
**World Bank: Global** **Food Safety Partnership**	x						x	x	
**Government of** **Cundinamarca**					x			x	
**Convenio Andres Bello**	x			x			x	x	x
**Public Universities &** **Primary and Secondary** **(K-11)**	x	x	x		x		x	x	
**Community, Farmers,** **Entrepreneurs, Food** **Vendors, Suppliers**	x		x		x	x			x

## Open Policy Recommendations

The adoption of an open policy represents a major culture change. For some it is a leap into uncharted territory. Although many organizations and businesses have successfully exploited the open ecosystem (see examples of success in Appendix A), in many respects it is easier to start something new in an open paradigm than it is to make the transition from a traditional approach. Historically, people have provided value through their proprietary content, process or service. As content, processes and services become available for free on the internet, basic assumptions and accepted business models are being challenged.

Recognizing that there is some information that should remain secured, there is probably more information and data that could be “freed” for mutual benefit than we currently realize. Providing seamless access to resources that could potentially be open while protecting those that need to be secured would be a breakthrough, forging a path for others to follow (and may lead to the creation of businesses around this new service). The GFSP is positioned to lead in this arena.

The World Bank adopted an Open Access Policy for Formal Publications as of July 1, 2012 which pertains to work carried out by Bank staff as well as outside research funded by the Bank.

At the GFSP meeting in Singapore Dec 10 – 11 ^th^, 2013 a preliminary proposed GFSP Openness Operating Principle was presented as follows:

“GFSP members intend to leverage existing knowledge, reduce duplicative efforts and speed and scale global solutions. One way GFSP members operationalize this intention is to enable the use, reuse, redistribution and remixing of the knowledge they choose to share as part of the partnership.”

To operationalize this intention, GFSP members agree to:

- use a standard creative commons copyright license to the extent possible.
- implement standard operating procedures for publication, such as editable file formats, standard file descriptions and publishing in web locations accessible to the public without a fee or registration requirements.”

The following broader statement is proposed to encompass the various facets of openness discussed in Appendix A. This will facilitate a staged approach as comfort with open concepts grows, without necessitating frequent revision of the policy.

In the interests of improving global food safety in a cost-effective and scalable manner, GFSP partners agree to proactively pursue “openness” as an operating principle. Partners will give consideration to the adoption and use of open strategies and tactics across all GFSP activities including:

- open content (including OERs and open courseware)
- open data
- open access (research)
- open government
- open source software
- open standards
- open policy
- open licensing
- open hardware

In so doing it is recommended that GFSP Partners:

- openly license all GFSP publicly funded deliverables with CC BY 4.0 license (or CC BY IGO 3.0 if IGO)
- develop GFSP deliverables in open file formats that are editable, customizable, and adaptable to local contexts
- establish a GFSP web site that makes GFSP openly licensed deliverables publicly available for free. Engage GFSP global network and public in use, reuse, and continuous improvement of openly licensed deliverables
- identify resources they currently have that could contribute to GFSP goals if openly licensed. Will bring forward those resources to the whole GFSP group and assess return on investment of combining those resources collectively across the partnership and with the new GFSP deliverables being developed.

GFSP partners have differing understanding of what openness is and what it means to global food safety and to their respective organizations. Organization of information sessions, workshops, events, activities and resources about openness would increase awareness, adoption and use both strategically and tactically.

To stimulate innovation in the adoption of open methods and the creation of new business models that leverage open methods GFSP partners should consider the use of open competitions either within specific projects or in general. Competitions are increasingly being utilized as a method to stimulate innovation and could be used to spur the adoption of open methods and the creation of open business models. Competition for grant funding has long been employed, however, in recent years variations such as the Gates Grand Challenges for Global Health (Archived at http://www.webcitation.org/6Y29b9N4L) and the XPRIZE competitions (Archived at http://www.webcitation.org/6Y2A0SkcW) have come into force. XPRIZE creates incentivized prize competitions “to bring about radical breakthroughs for the benefits of humanity, thereby inspiring the formation of new industries and the revitalization of markets”. A precedent for using competitions to encourage the use of open data has already been set by the HealthDataPalooza (Archived at http://www.webcitation.org/6Y2Ay4GJH) which hosts an annual competition for development of the best app using public health data. The competition is attended by several thousand people, including representatives from major health providers, venture capitalists and entrepreneurs and has led to the creation of new businesses. This could be replicated for food safety stakeholders.

The commitment to an open operating principle is founded on a belief that open strategies uniquely provide opportunities for the GFSP and all food safety stakeholders to disseminate and scale their work for greatest impact and global public good. Through openness the food safety community can leverage existing knowledge, reduce duplicative efforts, and speed and scale global solutions.

## Appendix A - Open practice definitions, examples of success, sample stakeholder value propositions

Open models utilize a range of open practices. Here are nine open practices, their meaning and their value proposition.

### 1. Open content (including Open Educational Resources/Open Courseware)

Open Educational Resources (OER) are teaching, learning, and research resources that reside in the public domain or have been released under an intellectual property license that permits their free use and re-purposing by others. OER include full courses, course materials, modules, textbooks, streaming videos, tests, software, and any other tools, materials, or techniques used to support access to knowledge (source: http://www.hewlett.org/programs/education/open-educational-resources Archived at http://www.webcitation.org/6Y2BJd5yH).

Examples of Success

Former Wall Street hedge fund analyst, Salman Khan, started creating web-based tutorials to help his cousin with her math which grew into the Khan Academy, reaching about 10 million students per month and delivering over 300 million lessons around the world. The Khan academy aims to provide “a free, world-class education for anyone, anywhere” and is one of the stellar examples of successful scaling using open educational materials.

Digital Green (Archived at http://www.webcitation.org/6Y2Bh9Pjy) is a knowledge platform helps farmers share best practices within their communities through production of digital videos. It allows farmers to see techniques demonstrated by their peers in their own language. The Food Safety Knowledge Network is an example of a University initiative providing free and openly licensed food safety learning resources that help stakeholders in emerging markets navigate the complexities of food safety and training.

Sample Stakeholder Value Propositions

Government entities, including regulatory agencies, ministries of health and human services, environmental services, etc. can provide a wide variety of training documents, generic food safety models, regulatory guidelines, memos, and other forms of open content that clarify and guide stakeholders in meeting regulatory requirements and food safety standards. There is significant value to all stakeholders in having a common authoritative reference point for all industry participants, minimizing the time and cost associated with acquiring accurate and consistent information regarding regulatory requirements and food safety standards.

NGOs play an important role in helping a wide variety of stakeholders better understand regulatory requirements, relevant food safety standards and policies, industry best practices, etc. Creating and releasing documents and other educational and informative content to the public as open content can extend the impact of the NGO in its mission.

Educational institutions have the potential to further their mission to educate the public and can work with faculty and staff to release course materials and other educational materials related to food processing, manufacturing, food management, food safety, etc. Furthermore, educational institutions and faculty can utilize open content to augment or add to gaps in curriculum, leading to opportunities for cost savings for the institution and students, as well as increased opportunities for knowledge sharing and collaboration.

It is within the interest of food manufacturers to provide suppliers with the knowledge they need to provide food products and other services that meet both the manufacturer’s standard of quality and the regulatory body’s expectation for food safety. Manufacturers, therefore, can provide a wide variety of food safety training documents, plans and procedures that suppliers can leverage to implement adequate training regimes. Providing open content also demonstrates a willingness to have additional public accountability and showcase a commitment to food safety. Additionally, there is potential for manufacturers and other stakeholders to leverage this content to form added value products and services, such as consulting and training services.

For new business owners or entrepreneurs, knowing where to begin in order to comply with food safety and regulatory compliance related issues can be a time consuming and costly undertaking. Access to open content, including food safety training documents, generic HACCP models and record keeping templates, regulatory compliance guidelines, etc. provides a valuable starting point for small and very small food manufacturers, vendors, and suppliers. This can result in significant cost savings for small business owners and also increase the likelihood that these entities attempt to abide by food safety standards and practices. Those who are successful in meeting compliance guidelines could gain credibility and possibly expand their business by documenting innovative and cost-effective means of compliance and sharing them with others. This might also be an efficient means of proving compliance to multiple buyers of their product.

### 2. Open Data

Open data is data that can be freely used, reused and redistributed by anyone, anywhere, for any purpose - subject only, at most, to the requirement to attribute and share-alike (summary of OpenDefinition.org). Historically very little open data has been available in areas such as health, energy, education, public safety, and global development. Today more and more of this data is becoming available and used by entrepreneurs, researchers, tech innovators, and others to create countless new applications, tools, services, and businesses.

Examples of Success

Several decades ago, the US government made a decision to make geographic information system (GIS) data publicly available and spawned a multi-billion dollar industry. In 2001 the first draft of the human genome was published in *Nature* ^iv^ as a result of international data sharing by researchers and its public release has led to the creation of hundreds of new drugs and new companies based on that data. In 2009 data.gov was created to make other types of US government data more accessible. In 2012, a national annual competition was created as part of the Health Data Initiative to stimulate the innovative use of health data in apps and products. The “Health DataPalooza” is now a sold out event attended by over 2,000 health providers, technology developers, venture capitalists, entrepreneurs and community advocates and has resulted in the launch of new products and companies. OpenFDA (Archived at http://www.webcitation.org/6Y2FDeT3K), providing easy access to public data of the US Food and Drug Administration (FDA) and highlighting projects using these data, was initiated in September of 2014.

Sample Stakeholder Value Propositions

Governments who collect data related to food safety, food industry trends, food safety research and analysis, can make these data open, leading to more informed decision making and the possibility of new business models that can build product and services offerings. These advances, ultimately, feed back into the economy.

Universities, colleges, and educational institutions engaged in activities and studies related to teaching or researching food safety, food manufacturing, food processing, management, etc. can release a wide variety of data collected about various fields that may enable stakeholders - particularly small businesses and entrepreneurs - to advance research, improve manufacturing or processing practices, develop added-value products or services.

Open data - whether historical data or current - can be analyzed and leveraged by small to medium enterprises to potentially design new technologies and software, assess particular market or industry trends, to learn about customer segments, and even develop practices that increase efficiencies in business operation and staffing, manufacturing, food processing, etc.

Many small and very small food businesses lack adequate resources to generate data that can be used as analysis tools to validate processes and substantiate food safety practices. Access to open data could enable these stakeholders to replicate processes and achieve food safety benchmarks. Furthermore, open data can be leveraged by the community and entrepreneurs to develop added value products and services that can have direct benefits to economies and businesses.

### 3. Open Access (Research)

By “open access” [to peer-reviewed research literature], we mean its free availability on the public internet, permitting any users to read, download, copy, distribute, print, search, or link to the full texts of these articles, crawl them for indexing, pass them as data to software, or use them for any other lawful purpose, without financial, legal, or technical barriers other than those inseparable from gaining access to the internet itself. The only constraint on reproduction and distribution, and the only role for copyright in this domain, should be to give authors control over the integrity of their work and the right to be properly acknowledged and cited (source: http://www.budapestopenaccessinitiative.org/boai-10-recommendations).

Examples of Success:

There are a number of open access journals and online publications that provide free and open access to scholarly articles specific to food safety, foodborne illness, manufacturing and processing practices, etc. In 2007 the US National Institutes of Health enacted an open access policy requiring the researchers they fund to make their final, peer-reviewed manuscripts publicly available no later than 12 months after official date of publication. The number of open access journals is rapidly increasing - the Directory of Open Access Journals lists over 9,000. The Public Library of Science (PLOS) and BioMed Central are two popular examples relevant to food safety.

Sample Stakeholder Value Propositions

Government agencies can provide and also promote (or mandate) access to publicly funded research and other food safety, processing, and scientific literature, which a wide variety of stakeholders can leverage in substantiating manufacturing practices. The public and economic value that stems from government agencies providing access to this information is far lower than the cost of correcting the public health problems and food safety risks that can stem from a lack of access.

NGOs involved in carrying out or sponsoring studies, research, and the publication of other scientific or relevant food safety literature can demonstrate a significant commitment to the dissemination of this literature through an open access policy, whereby content is freely available for people to freely access. The value to the organization in adopting this approach is in the potential for an increase in the organization’s reach and impact among key stakeholders and the opportunity to receive increased funding based on demonstrated impact. They can also leverage existing open access resources in the creation of additional knowledge and training resources intended for different audiences.

Universities and other educational institutions involved in publishing scientific and other literature related to food safety and food manufacturing can provide and promote free and open access this literature. A wide variety of stakeholders can leverage this literature and research to support food processing and manufacturing practices.

### 4. Open Government

Open government is the governing doctrine which holds that citizens have the right to access the documents and proceedings of the government to allow for effective public oversight ^v^. The Open Government Partnership currently involving 63 countries around the world has endorsed an open government declaration that commits them to, increase the availability of information about governmental activities, support civic participation, implement the highest standards of professional integrity, and increase access to new technologies for openness and accountability– (see more at: http://www.opengovpartnership.org/about/open-government-declaration).

Examples of Success

Open government initiatives are focused on a wide range of topics including access to information, anti-corruption, citizen participation, open data, and budget transparency. Success stories related to these topics can be found here. IPaidABribe.com (Archived at http://www.webcitation.org/6Y3QjqeNs), started by a non-profit in India, invites citizens to anonymously report the bribes they are asked to pay. It has over 20,000 bribe reports filed from 500 Indian cities and NGOs from 26 countries are interested in replicating its model.

Sample Stakeholder Value Propositions

By providing complete and open access to municipal, state, federal regulation, including guidelines, draft legislation, memos, discussion, generic food safety models, etc. government can demonstrate a commitment to transparency and public accountability. Constituents can subsequently provide input that helps guide legislation and guidelines, helping to preserve cultural or local practices and provide an understanding of how regulatory requirements may benefit or hinder the processes and practices of small and very small businesses.

### 5. Open Source Software

Open source software is software that can be freely used, changed, and shared (in modified or unmodified form) by anyone (Open Source Definition: opensource.org).

Examples of Success

Both new and established businesses have created valuable services around free software. Red Hat Inc. is a multinational software company founded in 1993 that provides open-source software products to the enterprise community. IBM is an example of an established multinational corporation that successfully incorporated the provision of open software support services into its business model and its employees actively contribute to open software development. Google and Apple are additional examples of corporations that have thrived by engaging the open source software developer community to build on their platforms.

Sample Stakeholder Value Propositions

The adoption of open source software can be usefully implemented to cut costs within a government organization and also boost innovation efficiency. A variety of technological product and service offerings can be built upon open source software with open application programming interfaces and open software development kits.

Educational institutions can both utilize and also support the development of open source software, including content and learning management systems, learning and other function based applications for mobile devices, and other computer based programs, that assist in helping both educate students and provide other stakeholders with tools and services directly applicable to managing and monitoring food processing and manufacturing operations.

### 6. Open Standards

Although there is no single definition for open standard (Archived at http://www.webcitation.org/6Y5L4eTYD), according to the French Government’s Law for Confidence in the Digital Economy it is understood to mean any communication, interconnection or interchange protocol, and any interoperable data format whose specifications are public and without any restriction in their access or implementation.

Examples of Success

Open standards in the technology space include the specification of open formats. The US Department of Labor published a set of guidelines for grantees in their Trade Adjustment Assistance and Community College Career Training grant program for use of open file formats in the development of OER. This ensures the OER can be easily edited and adapted by others.

Sample Stakeholder Value Propositions

All stakeholder entities have the capacity to establish both technical, communication, and document standards (including open document formats) to facilitate public access and exchange of information. Clear standards lead to greater interoperability and cost savings, which feed directly back into the economy.

### 7. Open Policy

Open policy involves organizations (including funders, government entities, non-profit organizations, education providers, and corporations) setting guidelines and policies that require the output of the research or work they fund or are involved with to be free and open to the public for use and repurposing. The underlying goal of open policy is to ensure that publicly funded resources are openly licensed and available to the public. Open policy can also pertain to resources created without public funding.

Examples of success

The World Bank supports the free online communication and exchange of knowledge as the most effective way of ensuring that the fruits of research, economic and sector work, and development practice are made widely available, read, and built upon. It is therefore committed to open access, which, for authors, enables the widest possible dissemination of their findings and, for readers, increases their ability to discover pertinent information. Open policy requires use of Creative Commons licenses (see full policy at: http://documents.worldbank.org/curated/en/2012/04/16200740/world-bank-open-access-policy-formal-publications).

The $2 billion US Department of Labor Trade Adjustment Assistance and Community College Career Training (TAACCCT) grant program has a policy that requires all grantees to license the new resources they create with grant funds using a Creative Commons Attribution license. This policy makes the TAACCCT program the biggest OER initiative in the world. A specific policy statement is found in solicitation for grant applications at: http://www.doleta.gov/taaccct/applicantinfo.cfm.

Sample stakeholder value propositions

Open policies significantly increase the amount and quality of publicly funded education, research, data, and software available to all.

### 8. Open licensing

Copyright law by default creates closed resources. Open licensing gives everyone from individual creators to large companies and institutions a simple, standardized way to grant permissions to their creative work while still maintaining copyright. Permissions typically pertain to the right to reuse, revise, remix and redistribute along with stipulations that allow commercial and/or non-commercial use and the requirement to share back adaptations. Open licensing requires downstream users to give attribution to the original creator. Open licenses for software and hardware work in a similar way.

Examples of success

Open licenses are used for software and for content. Openly licensed software includes, Linux, Moodle, DSpace, Android, and many other applications. In the content arena there are well over 500 million resources licensed with Creative Commons including all of Wikipedia, over 300 million photos on Flickr, and millions of videos on YouTube.

Sample stakeholder value propositions

Manufacturers and suppliers have the capacity to make significant inroads in building food safety awareness among suppliers, small and medium sized enterprises through the dissemination of training resources and other data, research, and literature related to meeting relevant food safety regulation. Providing these resources under open license such as Creative Commons licenses could provide a significant opportunity for organizations, innovators, and entrepreneurs involved in either food production or food safety education and training to develop product and service offerings for local or industry specific food safety related issues.

### 9. Open hardware

Open hardware are physical goods and technologies designed and offered through open design. Open hardware means that information about the hardware such as mechanical drawings, schematics, bills of material, layout data, and the software that drives the hardware, are all released with open licenses.

Examples of success:

See Open Source Ecology ( http://opensourceecology.org/) and Farm Hack ( http://farmhack.net) for examples relevant to food production and food safety. Photosynq (Archived at http://www.webcitation.org/6Y5LzH0vQ) is an open research project whose goal is to create a low cost, hand-held measurement device which researchers, educators and citizen scientists can use to build a global database of plant health. A low cost mobile prototype has been developed to replace the large, expensive and stationary equipment that was previously required to measure photosynthesis.

Sample stakeholder value propositions

Open hardware increases innovation by open collaboration making it possible to produce open source industrial machines and physical goods that can be made for a fraction of commercial costs.

### Open practice process

There is a graduated process in adopting open methods that most people go through, a set of steps or stages as follows:

1. Awareness - for most people openness is a new concept they aren’t even aware of.
2. Responding to and overcoming the initial fear reaction. Almost everyone initially expresses a great deal of fear over openness. Fears include issues such as remuneration, loss of livelihood, degradation of the resource and a negative effect on its initial integrity. An upside or opportunity must counteract and outweigh each fear.
3. Looking at examples. People want to see real examples of openness and hear the stories from those who have taken that path.
4. Trying it out. Once a certain level of comfort has been achieved people begin to see how others’ open work can benefit them personally. They dip their toe in and try using something that is open.
5. Going open yourself. Once you've sampled someone else's open work many then become willing to make their own work open - perhaps initially in a small way but gradually more and more.
6. Adopting open as a cornerstone of practice. Once you get to this stage you’re in all the way and usually become an advocate who won’t go back.
  Adopting open as a cornerstone of practice. Once you get to this stage you’re in all the way and usually become an advocate who won’t go back.
7. Spreading open. Someone who adopts open in one area (e.g. OER) then becomes interested in other areas of openness and starts to see the synergistic benefits of adopting more and more open practices. The cumulative benefits of multiple forms of openness are greater than each individually.

Usually one can’t move to stage 5, 6, or 7 without going through stages 1 through 4.

Another essential component of this is understanding the fundamental economic differences between digital and physical. Digital makes abundance possible. Physical is by its very nature a scarcity. Models based on abundance operate very differently than models based on scarcity. Abundance transfers power, knowledge and action from the few to the many.

## Appendix B - The economics of open and open business models recommended reading

Abel, B.V, Lucas Evers, Roel Klassen & Peter Troxler Ed. (2011). *Open Design Now*. Netherlands: BIS Publishers. Available for free under a Creative Commons license at: http://opendesignnow.org/

Anderson, C. (2010). *Free: How today's smartest businesses profit by giving something for nothing.* New York: Hyperion Books.

Benkler, Y. (2006). *The Wealth of Networks: How social production transforms markets and freedoms.* New Haven: Yale University Press. The author has made an online version of the book available under a Creative Commons Noncommercial Sharealike license; it can be accessed through the author’s website at http://www.benkler.org

Bollier, D. & Silke Helfrich Ed. (2012). *The Wealth of the Commons: A world beyond market & state.* Amherst: The Commons Strategies Group.

Jimenez, D., Amadeu Raya, Lluis Martinez, Irene Monsalve, (2010). *Economic Aspects and Business Models of Free Software*. Eureca Media: Barcelona. An openly licensed free downloadable version of this book is available online at the Free Technology Academy http://ftacademy.org/materials/fsm/5

Raymond, E. (2001). *The Cathedral and the Bazaar*. Sebastopol: O’Reilly Media.

Rifkin, J. (2014). *The Zero Marginal Cost Society: The Internet of things, the collaborative commons, and the eclipse of capitalism.* New York: Palgrave.

Stacey, P. (2012). *The Economics of Open*. Retrieved April 14, 2014. http://edtechfrontier.com/2012/03/04/the-economics-of-open/

Stacey, P. (2014). *New Models for a New Year*. Retrieved April 14, 2014. http://edtechfrontier.com/2013/12/29/new-models-for-a-new-year/
