# Supplementary material for: An open ecosystem engagement strategy through the lens of global food safety
Source: F1000Res. 2015 May 27;4:129. [Version 1] doi: 10.12688/f1000research.6123.1 (PMC4505781; doi:10.12688/f1000research.6123.1)
Supplement: Peer review history

# Peer review: Referee response for version 1

**DOI:** 10.5256/f1000research.6559.r9188

**Published:** 2015-07-06

**Authors:** Nikos Manouselis

**Affiliations:** 1 Agro-Know, Athens, Greece

This paper discusses how open models may be adopted by a traditional professional training ecosystem - the one of food safety. It explains the basic concepts around open models, explores examples of relevant open cases, and recommends some open policies that the particular ecosystem should follow in order to become more open. It also examines two specific cases of food safety training stakeholders: one in China and one in Colombia.

I found very interesting the fact that different types of open practices have been examined - including open data, content, government, software, hardware etc. This is providing a wide range of tools and interventions that can be adopted by the stakeholders in such an ecosystem, so that new business models may be explored. On the other hand, I believe that putting everything on the table in the case of such a traditional training market may be too radical and in a way intimidate stakeholders.

I would find it useful if the authors could reflect on this concern, possibly elaborating a bit more about a potential strategy that someone should adopt to introduce such novel models in a traditional training ecosystem. For instance, elaborating a bit on the Innovators and the Early Adopters that can help introduce such a radical innovation in the ecosystem, using ideas from approaches like the ones presented in the classic book Crossing the Chasm ( https://en.wikipedia.org/wiki/Crossing_the_Chasm).

Overall, a very solid submission and a novel contribution that provides some insight in the way that openness can be introduced in training markets.

I have read this submission. I believe that I have an appropriate level of expertise to confirm that it is of an acceptable scientific standard.

# Peer review: Referee response for version 1

**DOI:** 10.5256/f1000research.6559.r8944

**Published:** 2015-06-09

**Authors:** Ted Hanss Jr

**Affiliations:** 1 School of Information, University of Michigan, Ann Arbor, MI, USA

This is a concept paper proposing an "open ecosystem" approach for the World Bank-led Global Food Safety Partnership (GFSP). The title and abstract accurately and succinctly capture the aims of the paper, in which the authors advocate compellingly for abandoning current business practices and replacing them with open models across multiple strategies and tactics for the GFSP, with the aim of improving the ability of GFSP to meet its goals. Through relevant case examples and a thorough elucidation of open strategies (in Appendix A), the authors build the case for their argument. 

The authors make their case for content re-use by articulating how the GFSP will build on the efforts in China. Modification and re-use are key attributes of openly licensed content that have been well demonstrated and are documented in Appendix A. However, the case studies would benefit from sharing outcome measures, if available, from previous efforts that are relevant to the GFSP, such as demonstrating how open practices in the China example have magnified the reach of the training programs compared with proprietary approaches. 

Overall, the authors' recommendations follow logically from their analysis. The recommendations, while targeted at a specific World Bank effort, are generalizable, thus making the paper relevant to a wide audience.

I have read this submission. I believe that I have an appropriate level of expertise to confirm that it is of an acceptable scientific standard.
